# Fetal Posterior Fossa Anomalies: Diagnosis-Specific Ultrasound–MRI Concordance and Divergent Perinatal Outcomes

**DOI:** 10.3390/medicina62081460

**Published:** 2026-07-28

**Authors:** Raziye Torun, Hakan Golbasi, Mucahit Furkan Balci, Zubeyde Emiralioglu Cakir, Sevim Tuncer Can, Ilayda Gercik Arzik, Hale Ankara Aktas, Ilknur Toka, Atalay Ekin, Ozgur Oztekin

**Affiliations:** 1 Department of Perinatology, Izmir City Hospital, Izmir 35540, Turkey; drhkngolbasi@gmail.com (H.G.); atalayekin@hotmail.com (A.E.); 2 Department of Obstetrics and Gynecology, Izmir City Hospital, Izmir 35860, Turkey; 3 Department of Radiology, Tepecik Education and Research Hospital, Izmir 35020, Turkey

**Keywords:** posterior fossa anomalies, fetal MRI, prenatal diagnosis, prenatal ultrasonography

## Abstract

*Background and Objectives*: We aimed to examine the demographic characteristics, prenatal imaging findings, genetic evaluation results, and postnatal outcomes of fetuses with posterior fossa anomalies (PFAs), and also to evaluate the diagnostic concordance between prenatal ultrasonography (USG) and fetal magnetic resonance imaging (MRI). *Materials and Methods*: This descriptive study analyzed singleton pregnancies referred to Izmir City Hospital and Tepecik Training and Research Hospital for suspected fetal PFA between 2016 and 2024. Data including USG findings, fetal MRI reports, genetic results, and perinatal outcomes were extracted from institutional records. Diagnostic concordance between USG and MRI was statistically assessed using the kappa coefficient. *Results*: Out of 152 fetuses with suspected PFA on USG, 116 underwent fetal MRI. Following the exclusion of normal MRI findings (*n* = 23), the final cohort comprised 93 fetuses with confirmed PFA. Mega cisterna magna (MCM) was the most prevalent diagnosis (54.8%), followed by cerebellar hypoplasia (CH) (15.1%) and Dandy–Walker malformation (DWM) (10.8%). A moderate-to-good diagnostic concordance was observed between USG and MRI (kappa = 0.640, *p* < 0.001). Significant differences were noted across MRI groups regarding gestational age at diagnosis (*p* < 0.001) and birth weight, which was notably lower in CH compared to MCM (*p* = 0.004). Clinical outcomes varied significantly by diagnosis (*p* < 0.001); while 74.5% of MCM cases showed normal development, adverse outcomes predominated in CH and Walker–Warburg syndrome. *Conclusions*: Fetal PFAs are a heterogeneous group of anomalies with different diagnostic and variable prognostic profiles. Fetal MRI improves anatomical classification and provides clinically significant contributions to prenatal counseling and perinatal management.

## 1. Introduction

Fetal posterior fossa anomalies (PFAs) comprise a broad group of congenital central nervous system disorders affecting the cerebellum, vermis, and brainstem. This spectrum includes distinct entities such as Dandy–Walker malformation (DWM), vermian hypoplasia (VH), vermian agenesis (VA), Blake’s pouch cyst (BPC), and mega cisterna magna (MCM), each with its own embryological basis and variable prognostic implications [1].

Accurate prenatal identification of posterior fossa structures remains challenging. The cerebellar vermis undergoes dynamic development throughout pregnancy, and incomplete rotation or delayed maturation in the early second trimester can mimic pathological conditions. Furthermore, overlapping ultrasonographic features among different anomalies of the posterior fossa can further complicate differential diagnosis. These diagnostic challenges have contributed to ongoing debates regarding classification, nomenclature, and prognostic grouping [2].

Prenatal ultrasonography (USG) remains the primary imaging modality for evaluating the posterior fossa. Standard axial transcerebellar views allow measurement of the transcerebellar diameter and cisterna magna, whereas midsagittal views are essential for detailed assessment of the vermis, brainstem and fourth ventricle. Recent advances in targeted neurosonography, including high-resolution transvaginal imaging, assessment of intracranial translucency, and evaluation of the brainstem occipital bone distance, have improved the early detection of posterior fossa abnormalities [3,4].

Despite these advances, USG may still be limited in the evaluation of the posterior fossa because of fetal position, maternal body habitus, and acoustic shadowing from the calvarium. In particular, distinguishing between BPC, VH, and DWM on the basis of sonographic findings alone may be difficult. In such cases, fetal magnetic resonance imaging (MRI) can provide valuable complementary information through multiplanar assessment, superior soft-tissue contrast, and improved visualization of associated intracranial abnormalities [5,6,7]. Given the heterogeneity of PFA and the variability of reported outcomes, a comprehensive approach integrating prenatal imaging, genetic evaluation, and postnatal follow up is essential [8].

In this study, we evaluated the demographic characteristics, prenatal imaging findings, genetic assessments, and postnatal clinical outcomes of fetuses diagnosed with PFA. In addition, we aimed to assess the diagnostic agreement between prenatal USG and fetal MRI and to examine the impact of associated anomalies on postnatal prognosis.

## 2. Materials and Methods

This descriptive study included fetuses evaluated and diagnosed with PFA in the Perinatology Departments of Izmir City Hospital and Tepecik Training and Research Hospital between 2016 and 2024. The study protocol was approved by the Institutional Ethics Committee (approval number: 2024/201; approval date: 11 June 2024) and was conducted in accordance with the Declaration of Helsinki. Due to the retrospective design of the study, informed consent was waived. Data were obtained through review of the hospital database, perinatology USG reports, fetal MRI reports, genetic counseling records, invasive prenatal testing results, and neonatal and pediatric follow up files. Multiple pregnancies were excluded. For each case, fetal USG findings (including intracranial and extracranial findings), fetal genetic results, maternal demographic characteristics (age, gravidity, parity, and presence of systemic disease), and perinatal outcomes (pregnancy termination, intrauterine fetal death, delivery, and postnatal prognosis) were recorded.

Prenatal ultrasonographic examinations were performed using a Voluson E8 Expert system (GE Healthcare, Tiefenbach, Austria) equipped with a 2–9 MHz 2D curvilinear and a 9 MHz transvaginal transducer. USG examination was performed by maternal–fetal medicine specialists in accordance with ISUOG neurosonography guidelines, using transvaginal USG in vertex presentation and abdominal USG in breech transverse presentation, in cases with suspected anomalies following basic CNS examination [4].

PFAs were classified on the basis of prenatal imaging findings as follows [9]: DWM was defined by complete or partial agenesis of the cerebellar vermis, cystic dilatation of the fourth ventricle, and enlargement of the posterior fossa with upward displacement of the tentorium, torcula, and transverse sinuses. MCM was defined in cases with a normally formed vermis and a cisterna magna measuring more than 10 mm. BPC was considered in cases with an upwardly displaced but morphologically normal cerebellar vermis and preserved normal anatomy of the fastigium, tentorium, and cisterna magna. In addition, isolated VH and VA were defined as cases with normal posterior fossa size and anatomy but absent or underdeveloped cerebellar vermis. Cerebellar hypoplasia (CH) was diagnosed when the transcerebellar diameter measured below the fifth percentile for gestational age [10]. Walker–Warburg syndrome (WWS) was defined as a syndromic malformation pattern typically characterized by cerebellar and brainstem hypoplasia, a cobblestone cortex, and frequently associated ventriculomegaly or hydrocephalus [11].

Fetal MRI evaluations were performed within one week of the initial sonographic diagnosis. All scans were conducted in accordance with ISUOG guidelines using a 1.5-Tesla Siemens Aera scanner (Siemens Healthineers, Erlangen, Germany) equipped with a six-channel body coil. Examinations were conducted without maternal or fetal sedation and without the use of contrast agents, with the mother positioned either supine or in a lateral decubitus position. The standard imaging protocol included T2-weighted HASTE and TruFISP sequences with a slice thickness of 4 mm. When clinically indicated, T1-weighted FLASH sequences were additionally obtained. Diffusion-weighted imaging was performed in all cases, and B0 images were evaluated when necessary to assess for intracranial hemorrhage. All MRI studies were reviewed and interpreted by an experienced pediatric radiologist [12].

Diagnostic concordance was defined at the level of the specific PFA category. A case was classified as “confirmed on MRI” when the USG diagnosis was unchanged, and as “reclassified on MRI” when fetal MRI changed the USG-based category, either to a different PFA subtype or to a normal posterior fossa. No case represented a de novo anomaly detected only on MRI; all fetuses had a posterior fossa finding already suspected on USG.

Prenatal diagnosis was based primarily on USG findings and, in selected cases, was supported by fetal MRI and genetic testing. In live born infants, the diagnosis was reevaluated in light of postnatal imaging and genetic data. Neurodevelopmental assessment of surviving infants was performed using the Denver II Developmental Screening Test (Turkish standardization version) at a median postnatal age of 18 months (IQR: 12–24 months, range: 6–36 months). The tests were administered by certified pediatricians or a pediatric neurologist at the corresponding follow-up centers. Due to the retrospective design and reliance on institutional electronic registries, the examiners were not strictly blinded to the prenatal imaging and clinical history [13].

Invasive prenatal testing (amniocentesis or fetal blood sampling) was offered to all fetuses with a confirmed posterior fossa anomaly, regardless of whether associated intracranial or extracranial anomalies were present and regardless of family history or consanguinity. No clinical criterion was used to restrict the offer of testing. Following genetic counselling, 30 of 93 families (32.2%) accepted the procedure, while the remainder declined an invasive test; the principal reasons recorded for declining were the procedure-related risk of pregnancy loss, the perception that an invasive test was not warranted for an apparently isolated finding, and the fact that the result would not alter the parents’ decision regarding the pregnancy. Genetic analysis was performed in 30 fetuses. Karyotyping, chromosomal microarray analysis, and exome sequencing were applied according to the phenotype and the recommendation of the medical genetics department. Because uptake was determined entirely by parental decision, the tested subgroup may not be representative of the full cohort.

### Statistical Analyses

Statistical analyses were performed using IBM SPSS Statistics, version 30.0 (IBM Corp., Armonk, NY, USA). Continuous variables are presented as median (IQR) or mean ± standard deviation, as appropriate, and categorical variables as n (%). Comparisons of continuous variables across fetal MRI diagnostic groups were made using the Kruskal–Wallis test, followed by Bonferroni-corrected pairwise comparisons when applicable. Associations between categorical variables were assessed using the Pearson chi-square test. Agreement between prenatal ultrasonography and fetal MRI was evaluated using the kappa coefficient. A *p* value < 0.05 was considered statistically significant. Because several diagnostic categories included small numbers of cases, the corresponding subgroup analyses were interpreted cautiously.

## 3. Results

A total of 152 fetuses with a prenatal suspicion of PFA on USG were retrospectively evaluated. Fetal MRI could not be performed in 36 cases due to patient refusal. The documented reasons for refusal included maternal claustrophobia or anxiety in the closed magnet bore, persistent safety concerns regarding electromagnetic fields despite counseling, and logistical or travel constraints for patients referred from distant provinces. Among the remaining 116 fetuses who underwent MRI, 23 were reported as normal. Consequently, the final study cohort consisted of 93 fetuses with confirmed PFAs on fetal MRI.

The maternal and perinatal characteristics of the study cohort are summarized in Table 1. The median maternal age was 27 years (IQR 23–31). The median gestational age at diagnosis was 27 weeks (IQR 23–31). Median gravidity and parity were 2 (IQR 1–3) and 1 (IQR 0–2), respectively. The median gestational age at delivery was 38 weeks (IQR 37–40), and the median birth weight was 3050 g (IQR 2550–3500). Male fetuses constituted 67.7% of the cohort (*n* = 63), while female fetuses accounted for 32.3% (*n* = 30).

**Figure 1:**
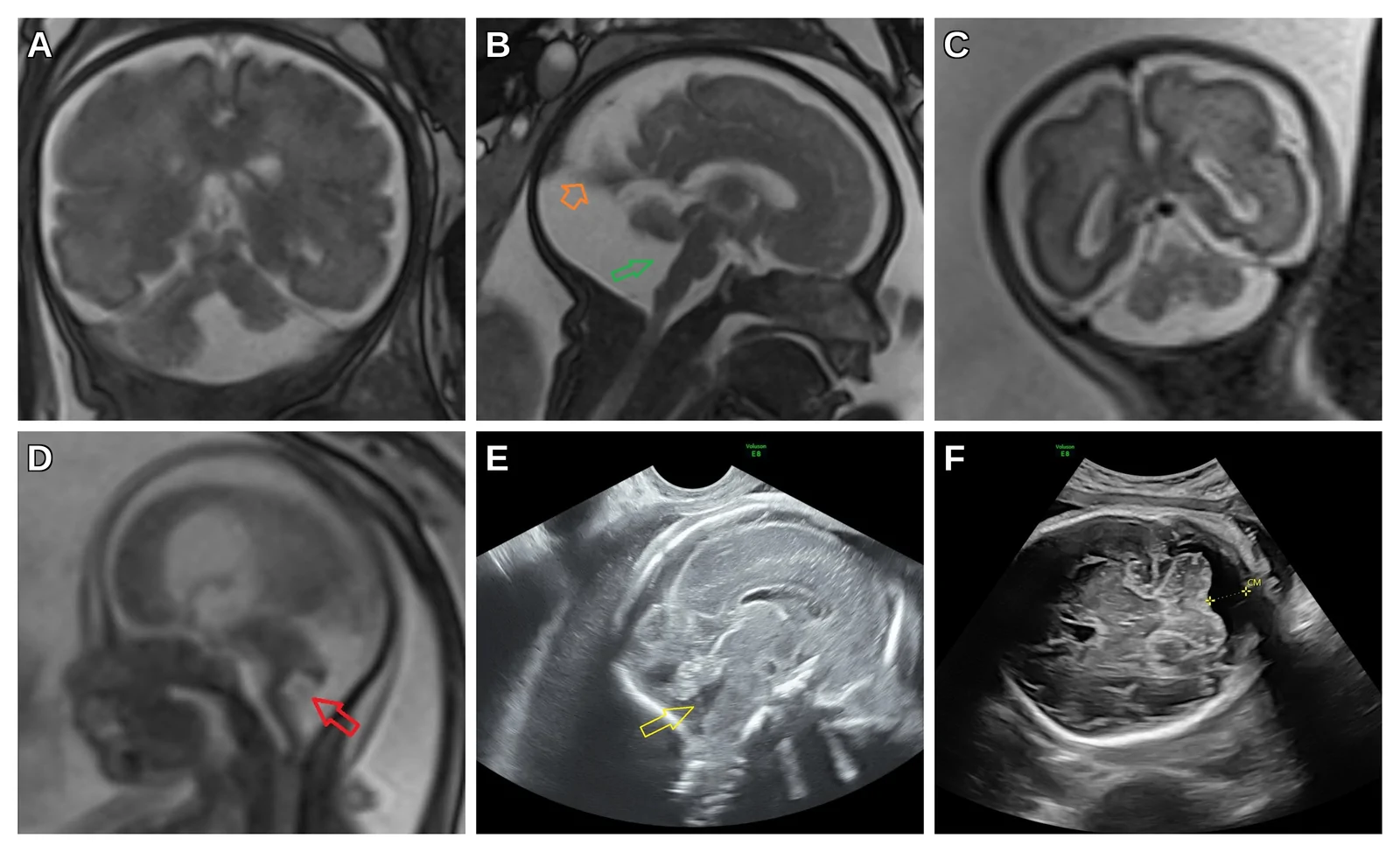
(**A**): Dandy–Walker malformation at 31 weeks. In fetal MRI, the separated cerebellar hemispheres are clearly visible in the transcerebellar section. (**B**): In the same case, torcular elevation (green arrow) and upward rotated hypoplastic vermis (orange arrow) are major characteristics of the anomaly. (**C**): Cerebellar hypoplasia at 31 weeks. (**D**): Walker–Warburg syndrome at 20 weeks shows cerebellar and brainstem hypoplasia (red arrow). (**E**): An increased tegmentovermian angle was observed on prenatal ultrasonography in a 23-week-old patient with a Blake’s pouch cyst (yellow arrow). (**F**): In a case of Mega Cisterna Magna at 28 weeks: prenatal ultrasonography revealed an enlarged cisterna magna in the transcerebellar axial plane.

PFA diagnoses according to prenatal USG and fetal MRI are presented in Table 2. In both imaging modalities, MCM was the most frequent diagnosis. Among the 116 fetuses who underwent fetal MRI, on prenatal USG, MCM was identified in 66 cases (57%), followed by CH in 17 cases (14.6%) and DWM in 9 cases (7.7%). On fetal MRI, MCM remained the most common diagnosis, detected in 51 cases (54.8%), followed by CH in 14 cases (15.1%) and DWM in 10 cases (10.8%) (Figure 1).

Concordance between prenatal USG and fetal MRI was evaluated in 116 cases (Table 2). The overall observed concordance was 74.1%, with a Cohen’s kappa of 0.640 (95% CI, 0.538–0.742; asymptotic SE, 0.052; *p* < 0.001), indicating moderate-to-good concordance between the two methods. The highest concordance was observed for DWM, WWS, and complete cerebellar agenesis (CCAg), whereas reclassification was more frequent in cases initially considered as VA, VH, and MCM. Of the 116 fetuses who underwent fetal MRI, the prenatal USG diagnosis was confirmed (concordant) in 86 (74.1%). In the remaining 30 cases, the diagnosis was reclassified on MRI: 23 were reclassified as a normal posterior fossa and excluded, while 7 were reassigned to a different PFA category. Accordingly, the final MRI-confirmed cohort of 93 fetuses comprised 86 concordant cases and 7 cases reclassified into a different PFA category.

Comparisons of maternal and prenatal variables across fetal MRI diagnostic groups are shown in Table 3. Gestational age at diagnosis differed significantly between groups (*p* < 0.001), whereas maternal age, gravidity, parity, and number of abortions did not. Post hoc analysis demonstrated that the MCM group had a significantly higher gestational age at diagnosis than the WWS, CH and DWM groups. Delivery outcomes were also compared by fetal MRI diagnosis. While gestational age at delivery did not differ significantly between groups (*p* = 0.153), birth weight showed a significant difference (*p* = 0.004). In post hoc analysis, birth weight was significantly higher in the MCM group than in the CH group.

Pregnancy and postnatal outcomes according to fetal MRI diagnosis are presented in Table 4. Overall, normal development was documented in 43 cases (46.2%), neurodevelopmental delay in 5 cases (5.4%), postnatal death in 15 cases (16.1%), intrauterine fetal death in 1 case (1.1%), and pregnancy termination in 10 cases (10.8%); outcome was unknown in 19 cases (20.4%). Outcome distribution differed significantly across fetal MRI diagnostic groups (χ^2^ = 86.006, df = 35, *p* < 0.001). MCM was associated with the most favorable outcome profile, with normal development observed in 74.5% of cases. In contrast, adverse outcomes were more frequent in the CH and WWS groups.

The distribution of associated anomalies and genetic findings is summarized in Table 5. Cranial anomalies were identified in 29 cases (31.2%), most commonly ventriculomegaly, followed by hydrocephalus and corpus callosum agenesis. Extracranial anomalies were present in 15 cases (16.1%), with gastrointestinal anomalies being the most frequent. Genetic results (karyotype, microarray, and exome analysis) were available in 30 cases, of whom 19 had normal findings. The remaining cases showed a range of chromosomal and monogenic abnormalities, including POMT1-related WWS, PTF1A mutation, trisomy 13, and chromosome 18 deletion.

When categorical variables were compared across fetal MRI diagnostic groups, significant associations were found for associated cranial anomalies (χ^2^ = 59.264, df = 30, *p* = 0.001; Cramér’s V = 0.64), genetic findings (χ^2^ = 68.346, df = 42, *p* = 0.006; Cramér’s V = 0.62), and fetal sex (χ^2^ = 23.350, df = 7, *p* = 0.001; Cramér’s V = 0.50), all indicating large effect sizes. No significant association was observed for associated extracranial anomalies (χ^2^ = 25.417, df = 24, *p* = 0.383) or mode of delivery (χ^2^ = 7.874, df = 7, *p* = 0.344). Among continuous prognostic variables, Spearman’s rank correlation revealed significant positive correlations between birth weight and both gestational age at diagnosis (ρ = 0.33, *p* = 0.005) and gestational age at delivery (ρ = 0.49, *p* < 0.001), whereas the correlation between gestational age at diagnosis and gestational age at delivery did not reach statistical significance (ρ = 0.22, *p* = 0.060). Male fetuses predominated in the MCM group, whereas female fetuses were more common in the CH group. In this cohort, all WWS cases had hydrocephalus and POMT1-related genetic abnormalities. Because several diagnostic subgroups contained small numbers of cases, these findings should be interpreted cautiously.

## 4. Discussion

In this descriptive study, we evaluated the distribution, prenatal diagnostic features, and perinatal outcomes of PFA confirmed by fetal MRI. MCM was the most frequent diagnosis, followed by CH and DWM. Fetal MRI showed moderate-to-good concordance with prenatal USG, but still led to diagnostic reclassification in a substantial proportion of cases, particularly in fetuses initially suspected of having MCM or vermian anomalies. Outcomes also differed considerably across diagnostic groups, with MCM showing the most favorable profile, whereas CH and WWS were associated with poorer outcomes.

Although the mean gestational age at diagnosis was 27 weeks, diagnoses were made across a wide range extending from the late first trimester to term. This likely reflects both the dynamic, gestational-age-dependent development of the posterior fossa and the heterogeneous nature of PFA. Consistent with previous reports, MCM was the most common diagnosis in our cohort and was generally identified at a later gestational age than other PFA [2,14].

Approximately three-quarters of fetuses in this group had normal postnatal development, whereas postnatal death and pregnancy termination were uncommon. This is consistent with previous reports suggesting that isolated MCM is generally a benign finding, particularly in the absence of associated intracranial or extracranial anomalies. These findings underscore the importance of distinguishing isolated cases from those with associated abnormalities during prenatal counseling [2,15,16].

In contrast, CH was associated with a clearly less favorable course in our cohort. This group showed lower birth weight, a higher rate of adverse postnatal outcomes, and relatively high rates of postnatal death and pregnancy termination. In addition, associated cranial abnormalities were common in these fetuses. These findings are consistent with the view that CH often represents part of a broader neurodevelopmental disorder rather than an isolated anatomical variant [17,18]. Previous studies have similarly shown that reduced cerebellar size, particularly when accompanied by other central nervous system abnormalities or genetic findings, is associated with less favorable neurological and developmental outcomes [19].

DWM accounted for a smaller proportion of the cohort than MCM but remained clinically important because of its heterogeneous presentation and outcome. In our series, gestational age at diagnosis was lower in the DWM group than in the MCM group, and half of the cases were associated with either postnatal death, pregnancy termination, or unknown outcome. This variability is not unexpected and likely reflects the well recognized heterogeneity of DWM, particularly with respect to associated anomalies and genetic background [2,14]. In some fetuses with a prenatal diagnosis of DWM, postnatal imaging may reveal a milder vermian abnormality, whereas others may show more complex brain involvement and unfavorable outcomes. Our findings therefore support the need for cautious prenatal counseling in this group, avoiding both overreassurance and overly pessimistic predictions while the anatomical picture is still evolving.

In our cohort, isolated VA and VH were frequently associated with ventriculomegaly and other intracranial abnormalities. Approximately half of these cases were followed by adverse perinatal or postnatal outcomes. These findings are consistent with previous reports suggesting that disorders of vermian development are often not truly isolated anomalies, but rather part of a broader neurodevelopmental spectrum [20,21].

Similar to MCM, BPC showed a relatively favorable profile compared with other posterior fossa anomalies, with no detected genetic abnormalities and only limited association with hydrocephalus or other major malformations. However, the number of BPC cases in our cohort was small, and those accompanied by ventriculomegaly were associated with unfavorable outcomes. These findings suggest that, although BPC is generally considered a benign entity, its prognosis may be adversely influenced by the presence of associated ventriculomegaly [22].

Despite the small number of cases, CCAg and WWS were consistently associated with severe outcomes in our cohort and generally carried a poor prognosis. Although the sample size was limited, the presence of hydrocephalus, genetic abnormalities, and adverse perinatal outcomes highlights the severity of these conditions and underscores the importance of early diagnosis in guiding prenatal counseling and clinical management [23,24]. In the present cohort, all cases of WWS were associated with hydrocephalus and POMT1-related abnormalities; because genetic results were available only for families who consented to invasive testing, this should be regarded as a descriptive observation consistent with the established literature rather than an independent genotype–phenotype association.

An interesting finding in our cohort was the significant variation in sex distribution across different PFA types. Specifically, male fetuses predominated in the MCM group, while female fetuses were more common in the CH group. The male predominance in MCM is consistent with established neurobiometric data showing that male fetuses naturally exhibit larger intracranial fluid volumes and wider cisterna magna dimensions during gestation. Because clinical diagnostic thresholds (e.g., cisterna magna width) are not adjusted for fetal sex, male fetuses are statistically more likely to be classified as having MCM. Conversely, the female predominance in CH may point to sex-specific genetic susceptibilities or potential male-lethal microdeletions in early embryogenesis, which warrant further investigation in larger molecular cohorts. Because several diagnostic subgroups were small, however, these observations should be regarded as hypothesis-generating.

A second important finding of our study is the added value of fetal MRI in the diagnostic evaluation of suspected PFA. Overall concordance between USG and fetal MRI was moderate to good, although reclassification still occurred in approximately one quarter of the cases examined. Diagnostic change was particularly evident in fetuses initially categorized on USG as having MCM or vermian abnormalities. In our cohort, fetal MRI did not reveal additional major adverse findings that resulted in a more severe reclassification; rather, it most often confirmed the initial ultrasonographic impression or improved anatomical delineation, thereby contributing to a more confident final diagnosis.

Its contribution likely relates to its multiplanar imaging capability and its improved delineation of associated intracranial findings. At the same time, the literature has raised concern about the possibility of overdiagnosis on fetal MRI in relatively subtle posterior fossa abnormalities, particularly inferior vermian hypoplasia [25]. For this reason, serial imaging and, whenever feasible, postnatal confirmation remain important. In our cohort, however, fetal MRI did not appear to lead to overdiagnosis; rather, in selected cases it clarified potentially overcalled sonographic impressions and allowed a more accurate final classification. These findings suggest that posterior fossa anatomy may be difficult to characterize reliably by USG alone, particularly in midgestation, and support the use of fetal MRI as a complementary tool in selected cases [7].

Associated anomalies also played an important role in our cohort. Cranial anomalies were present in nearly one-third of cases, and ventriculomegaly was the most common associated finding. Extracranial anomalies were less frequent, but still identified in a meaningful minority of fetuses. In addition, genetic results were available in approximately one-third of cases, and abnormal findings were detected in more than one-third of those tested. The significant associations observed between MRI diagnosis and both cranial anomalies and genetic findings further support the view that posterior fossa anomalies should not be considered in isolation. Rather, they should be interpreted in the broader context of fetal neuroanatomy, associated extracranial findings, and, when available, molecular testing. This is particularly relevant for prenatal counseling, since prognosis depends not only on the posterior fossa diagnosis itself, but also on whether the anomaly is isolated or part of a more complex syndromic process.

Our findings also have implications for prenatal counseling. The wide variation in postnatal outcomes across diagnostic groups indicates that a single counseling framework cannot be applied to all PFAs. The relatively favorable course observed in most fetuses with MCM differs clearly from the more severe pattern seen in CH, WWS, and some vermian anomalies. Accurate classification therefore remains central to meaningful counseling. In clinical practice, this means that prenatal counseling should rely on repeated targeted neurosonography, selective use of fetal MRI, and incorporation of associated anomalies and genetic data whenever possible. This further underscores the need for a multidisciplinary approach. Heterogeneity in prognosis and neurodevelopmental outcome depends on the specific type of PFA, whether it is isolated, and whether associated structural or chromosomal abnormalities are present. Accurate classification of PFA is therefore fundamental to prenatal counseling, as pregnancy and neonatal outcomes vary considerably according to the type of anomaly.

This study has several limitations. First, its retrospective design and incomplete data carry an inherent risk of inconsistent follow-up. Second, fetal MRI was not available in all fetuses with suspected PFA, mainly because of patient refusal, which may have introduced selection bias. Third, some diagnostic subgroups were small, limiting the robustness of subgroup comparisons and requiring cautious interpretation of categorical analyses. Furthermore, although invasive prenatal testing was systematically offered to all patients in our cohort, genetic data were available only for the 30 fetuses (32.2%) whose families consented to the procedure. This introduces a parental self-selection bias, as families facing more severe or complex prenatal diagnoses may have been more likely to accept invasive testing, and the true prevalence of chromosomal and monogenic abnormalities in fetal PFA therefore cannot be estimated from our data. Genotype–phenotype observations from this cohort, including the consistent association of WWS with POMT1 variants, should be regarded as descriptive observations within the tested subgroup rather than as generalizable associations. Additionally, 19 cases (20.4%) were lost to postnatal follow-up. This attrition is primarily attributed to the tertiary referral nature of our center; many families referred from distant provinces returned to their local healthcare providers for routine pediatric care after delivery, particularly in clinically stable or isolated cases (e.g., isolated MCM), so their long-term pediatric records were inaccessible to our institutional registry. Finally, although the Denver II test provided a validated and practical instrument for early neurodevelopmental screening, our follow-up data are limited by a relatively short median duration (18 months) and the lack of assessor blinding. Because subtle cognitive deficits, learning disabilities, or behavioral disorders may only manifest in later childhood, the high rate of ‘normal development’ observed in our MCM cohort (74.5%) must be interpreted cautiously, as it primarily reflects favorable early-childhood outcomes rather than long-term school-age performance. Despite these limitations, the study has several notable strengths. It covers a relatively long study period in a tertiary referral center, includes a clinically meaningful number of PFAs confirmed by fetal MRI, and integrates USG, fetal MRI, genetic data, and postnatal follow-up. In addition, the comparison between USG and fetal MRI provides information that is directly relevant to everyday clinical practice, particularly in settings where decisions regarding further imaging and prenatal counseling must be made in real time.

## 5. Conclusions

In conclusion, fetal PFAs represent a heterogeneous group of disorders with clearly distinct diagnostic and prognostic profiles. Prognosis depends largely on the specific diagnosis and the presence of associated structural or genetic abnormalities. In our cohort, isolated MCM and BPC were generally associated with favorable outcomes, whereas DWM, cerebellar agenesis, and syndromic conditions were linked to a substantially poorer prognosis. Fetal MRI showed moderate to good concordance with prenatal USG and provided additional value in selected cases by refining anatomical classification. Taken together, these findings support a stepwise diagnostic approach based on expert neurosonography, selective fetal MRI, thorough assessment of associated anomalies, and genetic testing when indicated. Accurate prenatal classification remains essential for appropriate counseling and perinatal management. Our data further indicate that reclassification is neither uniform nor unidirectional: concordance was complete for DWM, WWS, and CCAg but poor for vermian anomalies, and MRI more often corrected an overcalled sonographic impression than revealed unsuspected severe pathology. On the basis of these findings, we recommend reserving fetal MRI for cases with diagnostic uncertainty on neurosonography, particularly suspected vermian anomalies, DWM, or complex/syndromic malformations, rather than for all fetuses with suspected PFA. When indicated, fetal MRI is most informative in the late second or third trimester (generally after 24 weeks of gestation), when the cerebellar vermis and posterior fossa are more fully developed and the risk of misclassification (e.g., overdiagnosis of inferior vermian hypoplasia) is lower.

## Figures and Tables

**Table 1:** Maternal and perinatal characteristics of fetuses with PFA confirmed by fetal MRI (*n* = 93).

Variable	Value
Maternal age (years), median (IQR)	27 (23–31)
Gestational age at diagnosis (weeks), median (IQR)	27 (23–31)
Gravidity, median (IQR)	2 (1–3)
Parity, median (IQR)	1 (0–2)
Abortions, median (IQR)	1 (1–2) *
Gestational age at delivery (weeks), median (IQR)	38 (37–40) †
Birth weight (g), median (IQR)	3050 (2550–3500) †
Male fetus, n (%)	63 (67.7)
Female fetus, n (%)	30 (32.3)

Abbreviations: IQR, interquartile range. * Available for 25 cases. † Available for 71 cases. Continuous variables are presented as median (interquartile range), and categorical variables as number (percentage).

**Table 2:** Distribution of posterior fossa diagnoses on prenatal USG and fetal MRI, and diagnosis-specific concordance.

Diagnosis	Prenatal USG *n* (%) (*N* = 116)	Fetal MRI *n* (%) (*N* = 93)	Confirmed on MRI *n* (%)	Reclassified on MRI *n* (%)
DWM	9 (7.7)	10 (10.8)	9 (100.0)	0 (0.0)
MCM	66 (57)	51 (54.8)	50 (75.8)	16 (24.2)
CH	17 (14.6)	14 (15.1)	14 (82.4)	3 (17.6)
WWS	2 (1.7)	3 (3.2)	2 (100.0)	0 (0.0)
VA	11 (9.5)	5 (5.4)	4 (36.4)	7 (63.6)
VH	6 (5.2)	5 (5.4)	3 (50.0)	3 (50.0)
BPC	3 (2.6)	3 (3.2)	2 (66.7)	1 (33.3)
CCAg	2 (1.7)	2 (2.2)	2 (100.0)	0 (0.0)

Abbreviations: DWM, Dandy–Walker malformation; MCM, mega cisterna magna; CH, cerebellar hypoplasia; WWS, Walker–Warburg syndrome; VA, vermian agenesis; VH, vermian hypoplasia; BPC, Blake pouch cyst; CCAg: complete cerebellar agenesis. The “Fetal MRI” column is the final MRI-based diagnosis (denominator = 93) and is a marginal total, so it may exceed the “Confirmed on MRI” count when cases reclassified from another USG category were assigned to that diagnosis. Overall concordance 86/116 (74.1%); Cohen’s κ = 0.640 (95% CI 0.538–0.742; *p* < 0.001).

**Table 3:** Comparison of maternal and prenatal characteristics according to fetal MRI diagnostic groups.

Variable	DWM (*n* = 10)	MCM (*n* = 51)	CH (*n* = 14)	WWS (*n* = 3)	VA (*n* = 5)	VH (*n* = 5)	BPC (*n* = 3)	CCAg (*n* = 2)	*p*
Age (year)	27.5 (23.8–31.0)	26.0 (23.0–28.0)	28.0 (24.8–35.8)	27.0 (23.0–32.5)	31.0 (26.0–32.0)	23.0 (22.0–25.0)	28.0 (25.0–31.5)	33.0 (31.0–35.0)	0.099
Gestational age (week)	23.0 (21.2–23.8)	30.0 (27.0–34.0)	22.0 (21.0–25.8)	20.0 (18.0–20.5)	22.0 (22.0–23.0)	25.0 (21.0–30.0)	23.0 (21.0–23.0)	24.0 (20.5–27.5)	<0.001
Gravidity	3 (2–4)	2 (1–3)	2 (1–3)	2 (2–4)	2 (2–3)	2 (2–2)	2 (2–3)	4 (4–4)	0.525
Parity	2 (0–2)	1 (0–1)	1 (0–2)	1 (0–2)	1 (1–1)	1 (1–1)	1 (0–1)	2 (2–2)	0.839
Abortions *	1 (1–3)	1 (1–2)	1 (1–6)	2	1 (1–1)	1	2 (1–2)	2	0.723
Gestational age at delivery †	36.0 (35.0–39.0)	39.0 (38.0–40.0)	37.0 (36.5–39.0)	31.0	39.5 (39.2–39.8)	39.0 (38.5–40.5)	38.0 (37.5–38.5)	39.0	0.153
Birth weight (g) †	3300 (2650–3500)	3205 (2942–3615)	2300 (1775–2500)	2340	2875 (2688–3062)	2700 (2300–2780)	2415 (2222–2608)	3000	0.004

Abbreviations: DWM, Dandy–Walker malformation; MCM, mega cisterna magna; CH, cerebellar hypoplasia; WWS, Walker–Warburg syndrome; VA, vermian agenesis; VH, vermian hypoplasia; BPC, Blake pouch cyst; CCAg, complete cerebellar agenesis. Values are presented as median (interquartile range, 25th–75th percentile). Group comparisons were performed using the Kruskal–Wallis test. A *p* value < 0.05 was considered statistically significant. * Abortion data were available for 25 cases. † Gestational age at delivery and birth weight were assessed only in cases with available birth data (*n* = 71).

**Table 4:** Pregnancy and postnatal outcomes according to fetal MRI diagnosis.

Fetal MRI Diagnosis	Normal Development, *n* (%)	Neurodevelopmental Delay, *n* (%)	Postnatal Death, *n* (%)	IUFD, *n* (%)	TOP, *n* (%)	Outcome Unknown, *n* (%)	Total
DWM	1 (10.0)	0 (0.0)	3 (30.0)	0 (0.0)	1 (10.0)	5 (50.0)	10
MCM	38 (74.5)	4 (7.8)	1 (2.0)	0 (0.0)	0 (0.0)	8 (15.7)	51
CH	1 (7.1)	1 (7.1)	5 (35.7)	0 (0.0)	3 (21.4)	4 (28.6)	14
WWS	0 (0.0)	0 (0.0)	1 (33.3)	0 (0.0)	2 (66.7)	0 (0.0)	3
VA	1 (20.0)	0 (0.0)	1 (20.0)	1 (20.0)	2 (40.0)	0 (0.0)	5
VH	1 (20.0)	0 (0.0)	2 (40.0)	0 (0.0)	1 (20.0)	1 (20.0)	5
BPC	1 (33.3)	0 (0.0)	1 (33.3)	0 (0.0)	0 (0.0)	1 (33.3)	3
CCAg	0 (0.0)	0 (0.0)	1 (50.0)	0 (0.0)	1 (50.0)	0 (0.0)	2
Total	43 (46.2)	5 (5.4)	15 (16.1)	1 (1.1)	10 (10.8)	19 (20.4)	93

Abbreviations: DWM, Dandy–Walker malformation; MCM, mega cisterna magna; CH, cerebellar hypoplasia; WWS, Walker–Warburg syndrome; VA, vermian agenesis; VH, vermian hypoplasia; BPC, Blake pouch cyst; CCAg, complete cerebellar agenesis. IUFD, intrauterine fetal death; TOP, termination of pregnancy. Data are presented as n (%); percentages are row percentages within each diagnostic group. Group comparison: Pearson chi-square test (χ^2^ = 86.006, df = 35, *p* < 0.001). Because several subgroups contained small numbers of cases with sparse cells, the chi-square result should be interpreted with caution.

**Table 5:** Distribution of associated anomalies and genetic findings in the final cohort (n = 93).

Characteristic	*n*	%
Associated cranial anomaly		
Present	29	31.2
Absent	64	68.8
Specific anomalies (n affected) †		
Ventriculomegaly	17	18.3
Hydrocephalus	6	6.4
Corpus callosum agenesis	5	5.4
Schizencephaly	1	1.1
Arachnoid cyst	1	1.1
Microcephaly	1	1.1
Associated extracranial anomaly		
Present	15	16.1
Absent	78	83.9
Specific anomalies (n affected) †		
Facial	5	5.4
Cardiac	4	4.3
Gastrointestinal	6	6.5
Genitourinary	3	3.2
Genetic evaluation		
Genetic testing performed	30	32.2
Normal result	19	20.4
Abnormal result	11	11.8
POMT1-associated WWS	3	3.3
PTF1A mutation	3	3.3
Trisomy 13	2	2.1
Chromosome 18 deletion	1	1.1
Bartter syndrome	1	1.1
Ataxia–telangiectasia (ATM, 11q22.23)	1	1.1
No genetic testing	63	67.8

Data are presented as *n* (%), calculated from the total cohort (*N* = 93). † Subcategories are not mutually exclusive; some fetuses had more than one anomaly, so listed counts may exceed the number of affected cases.

## Data Availability

The data that support the findings of this study are available from the corresponding author upon reasonable request.
